# Endothelial progenitor cell-derived conditioned medium mitigates chronic cerebral ischemic injury through macrophage migration inhibitory factor-activated AKT pathway

**DOI:** 10.1186/s13287-024-04015-5

**Published:** 2024-11-14

**Authors:** Ya-Wen Cheng, Ling-Yu Yang, Yi-Tzu Chen, Sheng-Che Chou, Kuo-Wei Chen, Yi-Hsing Chen, Chuan-Rou Deng, I-Chin Chen, Wan-Ju Chou, Chen-Chih Chang, Yong-Ren Chen, Hsiao-Lin Hwa, Kuo-Chuan Wang, Meng-Fai Kuo

**Affiliations:** 1 Division of Neurosurgery, Department of Surgery, National Taiwan University Hospital, No.7, Chung-Shan South Road, Taipei, 100 Taiwan; 2 Graduate Institute of Clinical Medicine, National Taiwan University College of Medicine, Taipei, Taiwan; 3 Department of Internal Medicine, National Taiwan University Hospital, National Taiwan University College of Medicine, Taipei, Taiwan; 4 Non-Invasive Cancer Therapy Research Institute, Taipei, Taiwan; 5 Adjunct Visiting Staff, Division of Neurosurgery, Department of Surgery, National Taiwan University Hospital, Taipei, Taiwan; 6 Department of Obstetrics and Gynecology, National Taiwan University Hospital, Taipei, Taiwan

**Keywords:** Endothelial progenitor cell-derived conditioned medium, Cerebral ischemia, Macrophage migration inhibitory factor, AKT pathway

## Abstract

**Background:**

Chronic cerebral ischemia (CCI) is a significant health issue characterized by hypoperfusion due to damage or occlusion of the cerebral or carotid arteries. CCI may lead to progressive cognitive impairment that is considered as a prelude to neurodegenerative diseases, including dementia and Alzheimer's disease (AD). Endothelial progenitor cells (EPCs) have been implicated in vascular repair in ischemic cerebrovascular diseases, primarily by differentiating into endothelial cells (ECs) or through paracrine effects. However, the clinical transplantation of stem cell therapies remains limited. In this study, we investigated the effects of EPC-derived conditioned medium (EPC-CM) on the impaired vasculature and neurological function in a rodent model of CCI and the mechanism involved.

**Methods:**

EPC-CM was analyzed by cytokine array to identify key factors involved in angiogenesis and cellular senescence. The effects and mechanism of the candidate factors in the EPC-CM were validated in vitro using oxygen–glucose deprivation (OGD)-injured ECs and EPCs. The therapeutic effects of EPC-CM and the identified key factor were further examined in a rat model of CCI, which was induced by bilateral internal carotid artery ligation (BICAL). EPC-CM was administered via intracisternal injection one week post BICAL. The cerebral microvasculature and neurobehavior of the rats were examined three weeks after BICAL.

**Results:**

Macrophage migration inhibitory factor (MIF) was identified as a key factor in the EPC-CM. Recombinant MIF protein promoted angiogenesis and prevented senescence in the injured EPCs and ECs. The effect was similar to that of the EPC-CM. These therapeutic effects were diminished when the EPC-CM was co-treated with MIF-specific antibody (Ab). Additionally, the vascular, motor, and cognitive improvements observed in the BICAL rats treated with EPC-CM were abolished by co-treated with MIF Ab. Furthermore, we found MIF promoted angiogenesis and anti-senescence via activating the AKT pathway. Inhibition of the AKT pathway diminished the protective effects of MIF in the in vitro study.

**Conclusions:**

We demonstrated that EPC-CM protected the brain from chronic ischemic injury and promoted functional recovery through MIF-mediated AKT pathway. These findings suggest EPC-CM holds potential as a novel cell-free therapeutic approach for treating CCI through the actions of MIF.

**Supplementary Information:**

The online version contains supplementary material available at 10.1186/s13287-024-04015-5.

## Introduction

Chronic cerebral ischemia (CCI) is a chronic reduction in cerebral blood flow, leading to sustained hypoxia and nutrient deprivation in the brain. This condition is commonly associated with cerebrovascular diseases such as atherosclerosis, arteriosclerosis, hypertension, and various angiopathies [1]. The damages caused by CCI are global ischemic cerebral injuries, and the underlying pathological processes continue to progress over a prolonged period. The long-term cerebral hypoperfusion in CCI leads to the accumulation of oxidative stress and inflammation, which triggers neuronal apoptosis in selectively vulnerable regions of the brain [2]. Neurons that are critical for memory and learning are especially susceptible to ischemic damage. As a result, CCI primarily impacts cognitive functions, including deficits in memory and learning [3, 4], and can also contribute to motor dysfunction, mood disturbance, and eventually, vascular dementia or neurodegenerative conditions like Alzheimer’s disease (AD) [2, 5–7]. Addressing this issue and finding ways to support neuron survival and neurological recovery is crucial due to the global impact of cerebral ischemic diseases.

Endothelial progenitor cells (EPCs) are a small population identified in the bone marrow and peripheral blood that show high differentiation capacity. Studies demonstrated EPCs contribute to tissue repair and neovascularization, primarily through paracrine mechanisms or by differentiating into mature endothelial cells (ECs) [8, 9], which play an important role in ischemic diseases [10–12]. EPCs secrete a wide range of growth factors, chemokines, cytokines, and extracellular vesicles that contribute to angiogenesis, reconstruction of the vascular microenvironment [13, 14], and improve the recovery of neurological function after cerebral ischemic injury [15, 16]. Although cell-based therapy is promising in regenerative medicine, the administration of EPCs may have adverse side effects, including tumorigenicity, low survival rate of the transplanted cells, and the immunogenic potential [17]. Therefore, conditioned medium (CM) collected from EPCs has emerged as an alternative potential cell-free therapeutic agent [18–20].

Our previous study demonstrated that the administration of EPCs combined with indirect revascularization protected against chronic cerebral ischemic injury [21]; however, the therapeutic effects of the secretory factors from EPCs have not been investigated in preclinical models of CCI. Here, we investigated the therapeutic effects of EPC-derived CM (EPC-CM) on CCI and the molecular mechanism involved in the vascular and neural repair process. Many proteins and cytokines are secreted from EPCs, and we focused on macrophage migration inhibitory factor (MIF) among the secretory factors [22]. MIF is a pleiotropic protein secreted by different types of cells, including neural stem/progenitor cells and EPCs [23, 24]. In addition to being recognized as a regulator of the immune response, increased studies have demonstrated that MIF is involved in a variety of signaling pathways which are important for the maintenance of cellular homeostasis, such as promoting cellular survival, angiogenesis, and anti-senescence [24, 25].

In this study, a CCI animal model was established by bilateral internal carotid artery ligation (BICAL) in male Wistar rats to investigate the therapeutic effects of EPC-CM. An in vitro cell-based system was used to study the molecular mechanisms of EPC-CM and MIF that may be involved in the vascular and neuronal repair process. The results demonstrated that EPC-CM can ameliorate ischemic injury and promote vascular and neuronal repair in the brain via MIF-mediated AKT pathway activation.

## Methods

### Cell experiments

#### Isolation and culture of endothelial progenitor cells (EPCs)

The EPCs were isolated from the human umbilical cord blood obtained from healthy volunteers after birth according to the previous study [21]. This study was approved by the Institutional Review Board of the National Taiwan University Hospital, Taipei, Taiwan, with the consent obtained from all of the donors. Briefly, the mononuclear cells (MNCs) in donors’ umbilical cord blood were isolated using Ficoll-Paque PLUS (GE Healthcare) density centrifugation, followed by washing with 1 × phosphate-buffered saline (PBS). 8 × 10^6^ isolated MNCs plated in 100-mm Petri dish pre-coated with fibronectin (10 μg/mL) and incubated with endothelial cell basal medium-2 (EBM-2) (Lonza) supplemented with endothelial cell growth medium-2 (EGM-2) MV SingleQuots (Lonza) containing 20% FBS. Cells were cultured at 37 °C with 5% CO_2_ and the medium was changed every 3 days until completion of differentiation had been established by morphology. According to previous studies, we used the late EPC-derived population, which is characterized by flow cytometry and immunofluorescence staining for the expression of CD34, VE-cadherin, CD31, vascular endothelial growth factor receptor 2 (VEGFR2), and CD133, as the source for conditioned medium, and also for the experiments in our study (Additional file 1: Fig. S1 A, B) [21, 26].

#### Preparation of serum-free EPC-derived conditioned medium (EPC-CM)

To collect the conditioned medium (CM), 80% confluent young EPCs (passage 2–5, without senescence signal, Additional file 1: Fig. S1 C) were cultured in 150 cm^2^ cell culture flask (Corning) and incubated with 10 ml serum-free DMEM nutrient mix F12 (Gibco) and 10 ml Hanks' balanced salt solution (HBSS) contained 200 μl P/S (1%) for 12 h. The medium was collected and concentrated by a Tangential Flow Filtration (TFF) membrane filter system (Millipore) unit with a 30 kDa cut-off (Millipore) following the manufacturer’s instructions (Concentration level: 10X). The filtered and concentrated medium was used as the EPC-CM in this study. Fresh endothelial medium (serum-free DMEM/F-12: HBSS = 1: 1) was directly filtered and concentrated as the basal medium that used for control and OGD (Mock) treatment in in vitro experiments.

#### Cytokine arrays of EPC-CM

Cytokine profile in the EPC-CM was determined using Quantitative Cytokine Quantibody Human Array 4000 (RayBiotech, Norcross, GA) following the manufacturer’s protocol. The array was similar to traditional sandwich-based ELISA, except that capture antibodies for a number of protein targets were attached to a glass slide in an array format which allowed for the multiplex detection of more proteins in one sample at one time. The expression levels of 200 soluble human proteins were determined in the Quantibody Human Array 4000 and listed at Additional file 1: Table S1.

#### Human umbilical vein endothelial cells (HUVECs) and oxygen–glucose deprivation (OGD) treatment

HUVECs were purchased from the Bioresource Collection and Research Center (BCRC) in Taiwan. Cells were cultured and maintained in EBM-2 (Lonza) supplemented with EGM-2 MV SingleQuots (Lonza) containing 10% FBS and 1% penicillin/streptomycin (P/S; Gibco) and incubated at 37 °C and 5% CO_2_ in a humidified atmosphere. To proceed the OGD treatment, the medium was replaced with 5% FBS medium with 80% confluence of the cells and transferred to the anaerobic chamber (Billups-Rothenberg Inc.) for 24 h in 1% oxygen. The EPC-CM group was incubated with 5% FBS medium with 20% EPC-CM; the other groups were supplied with 5% FBS medium with 20% basal medium, while the normoxic group was maintained in the complete medium with 20% basal medium. Next day, the cells were collected for Western blotting or transferred to normoxic, 37 °C and 5% CO_2_ incubator for further experiments.

#### Western blotting analysis of signaling pathways in cells

40 µg protein extracted from the cultured cellular lysates was separated by 12% sodium dodecyl sulfate–polyacrylamide gel (SDS-PAGE, Bio-Rad) and transferred to polyvinylidene fluoride membranes (PVDF, Bio-Rad). Membranes were blocked in 5% non-fat milk for 1 h and hybridized overnight at 4 °C with the following primary antibodies: rabbit monoclonal phospho-AKT (Ser473) (1:1000, Cell Signaling), rabbit monoclonal pan-AKT (1:1000, Cell Signaling), rabbit polyclonal phospho-p44/42 MAPK (ERK1/2) (Thr202/Tyr204) (1:1000, Cell Signaling), rabbit polyclonal p44/42 MAPK (ERK1/2) (1:1000, Cell Signaling), and rabbit monoclonal MIF (1:1000, Abcam). GAPDH (1:10,000, Thermo fisher) was used as a loading internal control. The secondary antibodies used in this study were donkey anti-rabbit (1:5000, Gene-Tex) and anti-mouse antibody (1:5000, Gene-Tex) conjugated with horseradish peroxidase (HRP). Signals were visualized using the enhanced chemiluminescence (ECL, Amersham) and imaged by the Diversity One software package (PDI, NY, USA).

#### Tube formation

The angiogenesis ability of ECs was evaluated by processing the tube formation assay. 5 × 10^5^ HUVECs were seeded in the 6-well plates for 24 h. The next day, the medium was replaced with 5% FBS contained EGM-2 medium with basal medium (20%) or EPC-CM (20%) or recombinant MIF (100 ng/ml) or PI3K/AKT inhibitor (5 µM LY294002) treatment, and cells were exposed to hypoxic incubator (1% O_2_, 5% CO_2_, and 94% N_2_) at 37 °C for 24 h to mimic chronic ischemic injury. The cells treated with 20% basal medium remained in the humidified atmosphere of 5% CO_2_ at 37 °C as the normoxic control. After 24 h, 5 × 10^4^ cells were suspended and reseeded into Matrigel (Corning Inc., USA)-coated 24-well plate for 4 h. Tube formation was observed with microscopy, and five independent fields were assessed for each well and acquired using a Nikon Eclipse Ti2 fluorescence microscope attached to a digital camera and Nikon NIS Elements imaging software. The total tube length of the images was analyzed by MetaMorph Premier Offline.

#### Cell migration assay

To evaluate the cell migration ability mediated by EPC-CM. 80% confluency of cells in the 6-well plates treated with different reagents as mentioned previously were incubated in the hypoxic incubator (1% O_2_, 5% CO_2_, and 94% N_2_) at 37 °C for 24 h. Control cells treated with 20% basal medium remained in the normoxic humidified atmosphere of 5% CO_2_ at 37 °C. After 24 h, cells were suspended and seeded into the transwell chamber (8 µm, Life science) with 300 µl serum free EGM-2 and inserted into a 24-wells plate. The lower chamber added with 500 µl EGM-2 medium contained with 5% FBS with basal medium (20%) or EPC-CM (20%) or recombinant MIF (100 ng/ml) or PI3K/AKT inhibitor (5 µM LY294002) treatment. The lower chamber contained EGM-2 medium with 10% FBS as normoxic control. After incubation in the humidified atmosphere for 24 h, removing the cells on the upper chambers by using a cotton swab, and cells adhered to the lower side of transwell were fixed with 4% paraformaldehyde, subsequently stained the cells with 4’,6-diamidino-2-phenylindole (DAPI) for 10 min, followed with 0.5% crystal violet staining for 10 min. The migrated cells were imaged and counted for five independent fields for each well by using a Nikon Eclipse Ti2 fluorescence microscope attached to a digital camera and Nikon NIS Elements imaging software.

#### Senescence induction and detection

2 × 10^5^ young HUVECs or EPCs (passage < 10) were seeded in 12-well plates for 24 h. Next day, cells were treated with EGM-2 contained 5% FBS and H_2_O_2_ (200 μM) for 2 days. The medium was then removed and changed to 5% FBS EGM-2 contained either 20% basal medium with/without rMIF (100 ng/ml) or 20% EPC-CM for 3 days. The cells were fixed and detected by a Senescence-associated (SA)-β-galactosidase Staining Kit (Merck) according to the instruction manual. The cells were then incubated at 37 °C for 16 h and stained with DAPI for the nuclear staining. The SA-β-gal-positive cells were observed by microscopy, and counted in five independent fields. The percentage of SA-β-gal-positive cells was normalized with the total cell numbers.

### Animal experiments

This animal experiments were conducted in accordance with the ARRIVE guidelines 2.0. Animals were kept three per cage in a constant 12 h light/dark cycle, at room temperature (21–25 °C) and humidity (45–50%) with free access to food and water.

#### Animal model of chronic cerebral ischemia (CCI): bilateral internal carotid artery ligation (BICAL)

Male 6-week-old Wistar rats (200–250 g, body weight) were randomly assigned to different treatment groups. There were four groups in this study: control, BICAL, EPC-CM treatment, and MIF Ab treatment groups. The total animals used in this study were 80 rats that were randomly and equally divided to control and each experimental group based on our previous experience [21]. Animals were anaesthetized by intraperitoneal injection of Zoletil (50 mg/kg) and Xylazine (8 mg/kg) to process surgery. Animals were also administered atropine sulfate (0.05 mg/kg, i.p.) to reduce hypersalivation. The BICAL procedure was performed as described previously [21]. Briefly, a longitudinal incision was made in the midline of the neck, and the bifurcation of the common carotid artery (CCA) was carefully dissected. The internal carotid artery (ICA) distal to the bifurcation of CCA was ligated with 6–0 silk suture. To decrease mortality of the experimental rats, ligation on the opposite ICA was performed 30 min later. The skin was then closed. The mortality rate after BICAL was 11%, and these rats were excluded from the experimental analysis. Sham surgery underwent the same procedure without ligation of bilateral ICAs. Three weeks after BICAL, the rats were deeply anesthetized with an intraperitoneal injection of Zoletil (50 mg/kg) and Xylazine (8 mg/kg), followed by euthanasia through exsanguination. Throughout the entire study, the researchers were blinded to the treatment allocation.

#### Treatment algorithm

The optimal dose of EPC-CM was determined by administering either a low dose (20 µL/rat) or a high dose (40 µL/rat) in BICAL rats, as has been performed in a previous study [27]. The high dose of EPC-CM showed significant improvement on the motor and cognitive functions in BICAL rats, but the low dose did not (Additional file 1: Fig. S2). Consequently, the high dose (40 µL/rat), administered at a slow rate of 40 µL over 30 s, was selected for treatment in this study. Briefly, the control and BICAL animals received an injection of 40 μl vehicle, i.e., 0.9% normal saline into the cisterna magna, while the treatment groups received intracisternal injection of EPC-CM (40 μl) or EPC-CM + MIF Ab (anti-MIF antibody 2 μl mixed with 40 μl EPC-CM) 1 week after BICAL procedure. The body temperature was maintained stable for 3 h after the intracisternal injections.

#### Microvasculature density

Animals were fixed in a stereotactic frame after anesthetization. We performed a 3 × 3 mm^2^ craniotomy behind the left coronal suture and removed the dura mater carefully without causing injury to the brain surface. The microcirculation on the brain surface was evaluated using a CAM1 capillary anemometer (KK Technology, UK) with a high-resolution (752 × 582 pixels) monochrome charge-coupled device (CCD) video camera. The microvasculature density was calculated using the De Backer's score [28], which was calculated as the number of vessels crossing a line divided by the total length of the line. A cut-off point of 20 µm was used to differentiate between small vessels and large vessels. For each rat, 5–10 images (640 × 480 pixels) were captured and each image was divided into 6 × 4 lines. The numbers of vessels crossing each line were counted and the sum was obtained from these images. The average number of crossing points was then calculated to determine the density of microvasculature.

#### Regional brain blood flow and brain tissue oxygen tension

The OxyLite 2000E and OxyFLO 2000E detectors (Oxford Optronic Ltd, England) with a fluorescence quenching technique were used to measure regional brain blood flow and partial pressure of brain tissue oxygen (PbtO_2_) in rats. To position the detection probe, the rat and probe were fixed to a stereotactic apparatus and the probe was placed 2 mm deep from the brain surface. PbtO_2_, blood flow, and temperature of the same micro-region were continuously monitored for 30 min.

#### Rotarod performance test

The rotarod evaluates motor coordination, balance, and endurance, which assess motor deficit in animal model [29]. Rotarod test was performed as previously described [28]. Rats were pre-trained for 3 days at 4 rpm, 3 sessions per day for 5 min on the instrument (Panlab Rota Rod, Harvard Apparatus) before BICAL surgery. Three weeks after BICAL, the rats were placed on the instrument and the latency to fall was measured under continuous acceleration (4 to 40 rpm for 600 s) by observers blinded to group assignment.

#### Open field test (OFT)

OFT is often used to measure general locomotor activity and anxiety-like behavior. Three weeks after BICAL procedure, the animals were put in an open-field apparatus (60 × 60 × 100 cm in dimension) and a video camera was equipped above the apparatus to record each 5 min trial. The mean overall distance traveled on the heat map of each animal was recorded and analyzed using the EthoVision XT 17.0 tracking system.

#### Novel Object Recognition (NOR) test

NOR test was conducted to assess short-term recognition memory of the animals [30]. The NOR testing chamber consisted of a black Plexiglas box (59 × 59 cm) with black walls that were 40 cm high. To analyze the test phase, a video camera was positioned above the chamber to record the activity. During the habituation phase, each rat was allowed to explore the empty arena freely for 5 min for 3 consecutive days. Three weeks after the BICAL surgery, rats were allowed to explore two identical objects (A + A) positioned symmetrically within the arena for 5 min to become familiar with the objects. Then the rat was placed back in the home cage, after which the test trial was conducted 1 h following the familiarization phase. During the test trial, one of the previously familiar objects (A) was substituted with a novel object (B) of a distinct material, shape, and color. Each animal was placed back into the arena for exploration of both objects (A + B) with 5 min. To prevent odor recognition, the objects and the box were thoroughly cleaned with 70% ethanol between each trial. Heat map analysis of animal tracking and the duration of time spent with each object were measured using a tracking system (EthoVision XT 17.0). Object exploration was operationally defined as the rat actively exploring an object while maintaining a distance of less than 2 cm with nose touching it. Discrimination of recognition novelty was quantified using a preference index: (time exploring the new object − time exploring the old object) / (total time exploring an object).

#### Y-maze test

The Y-maze spontaneous alternation test was used to assess spatial working memory, as described previously [31]. Three weeks after BICAL surgery, rats were placed at the end of one arm (labeled “a”) and allowed to move freely through the Y-maze (a, b, c arms) and monitored with an 8 min period with a video tracking system. Spontaneous alternation performance (SAP) was only counted when rat entered three different arms consecutively (e.g. abc, acb, bca, bac…). The spontaneous alternation (%) was calculated as the percentage of [(number of alternations) / (total arm entries − 2)] × 100.

#### Immunofluorescence for vessels labeling

For immunofluorescence staining, brain sections were permeabilized with 0.1% Triton X-100, blocked in 5% BSA for 1 h at room temperature, followed with incubated with the Lycopersicon esculentum (tomato) lectin (LEL, TL) antibody (Thermo Fisher), a widely used endothelial marker for vessels labeling [31], at room temperature for 1 h. The nuclei are counterstained with DAPI (Invitrogen). Mounted slides subjected to analysis through digital fluorescent microscopy and the quantitative analysis of vessel density was performed using ImageJ software (NIH, USA).

### Statistical analysis

Data collection and analysis were carried out by investigators who were blinded to the experimental conditions. Data were expressed as the mean ± standard deviation (SD). Data were analyzed for normality by the Shapiro–Wilk normality test. One-way analysis of variance (ANOVA) followed by a Post Hoc Test (Tukey’s multiple comparisons test) was used for normally distributed data. Bar graphs and statistical analysis were performed by GraphPad Prism 7.0 software (GraphPad Software, USA). A P-value of < 0.05 was considered a statistically significant difference.

## Results

### EPC-CM and MIF protein promote angiogenesis in OGD-treated ECs

**Fig. 1:**
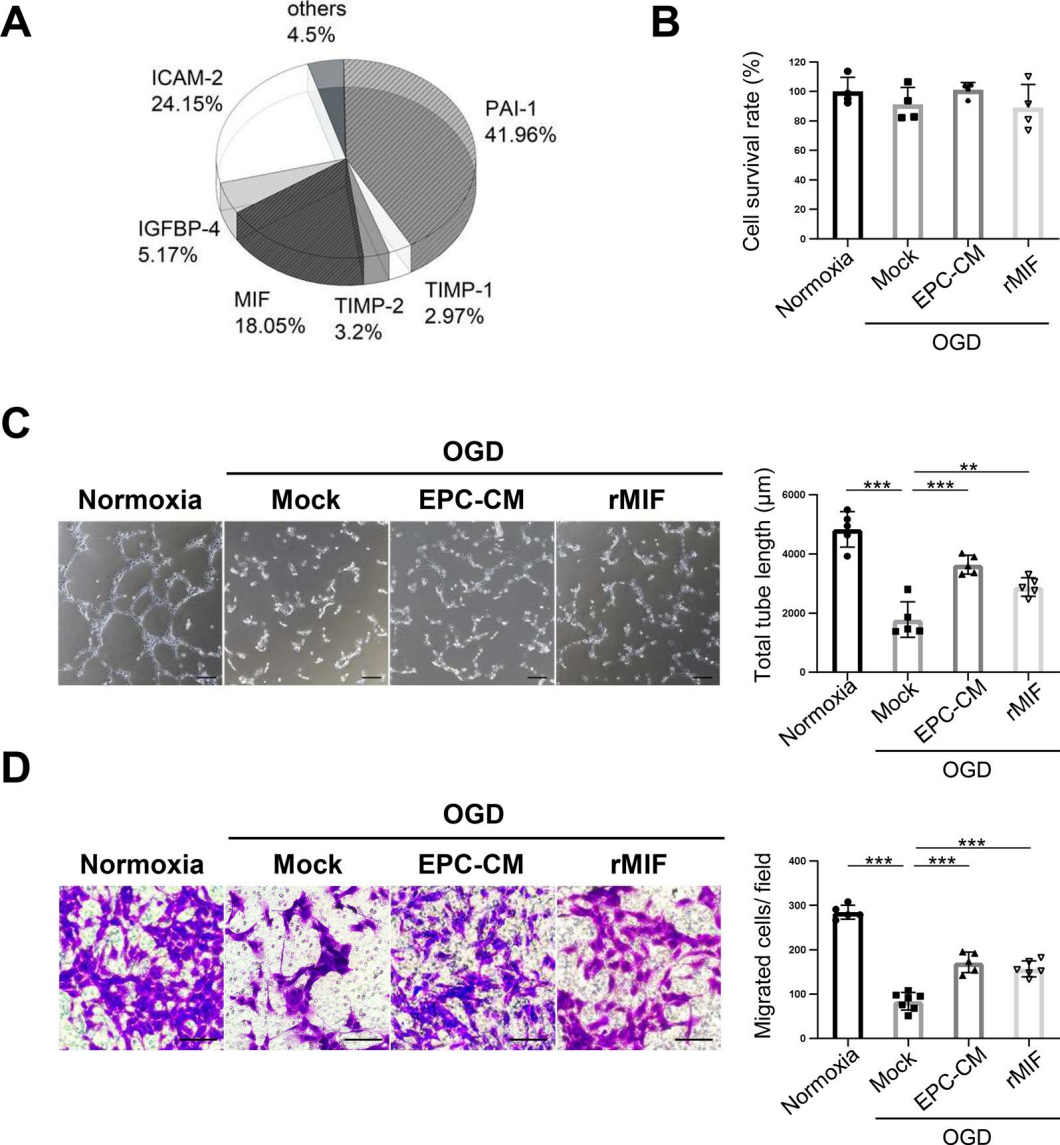
EPC-CM and MIF protein promote the angiogenesis ability in OGD-treated ECs. **A** Cytokine profile in the EPC-CM was determined using Quantitative Cytokine Quantibody Human Array 4000 (RayBiotech, Norcross, GA). **B** The cell survival ratio of the OGD-treated HUVECs having treated with different compounds (Normoxia: 20% basal medium, OGD/Mock: 20% basal medium, EPC-CM: 20% EPC-CM, rMIF: 20% basal medium with 100 ng/ml rMIF) was detected by CCK-8 assay and normalized with normoxic group. **C** Left panel, representative images of the tube formation in HUVECs with different treatments described in (B). Right panel, the average total length of tubes was quantitatively analyzed by 5 fields randomly. Scale bar = 100 μm. **D** Representative images of the crystal violet staining of the cells passed through the transwell membrane after different treatments described in (B) (left panel). Scale bar = 100 μm. The migrated cell numbers in each group were quantitatively analyzed by 6 fields randomly (right panel). The data was shown as the mean ± SD and analyzed by One-way ANOVA. ** *p* < 0.01, *** *p* < 0.001

A cytokine array analysis of EPC-CM was conducted to identify the potential critical factors in EPC-CM involved in mediating neovascularization and vascular repairs during ischemic events. The analysis identified MIF as a potential candidate, which plays important role in promoting angiogenesis and cell survival [32, 33] (Fig. 1A, Additional file 1: Table S1). To understand the regulatory role of MIF in EPC-CM during ischemic events, we examined its effect on the function of ECs under OGD conditions, a widely used in vitro ischemic model [34]. HUVECs treated with/without EPC-CM or recombinant MIF protein (rMIF) then subjected to OGD to mimic chronic ischemic conditions. After incubating HUVECs under OGD (1% O_2_) for 24 h, cell survival was assessed to evaluate OGD-induced damage. The data showed no significant differences between each group, suggesting that the OGD conditions did not cause acute damage (Fig. 1B). Given the importance of neovascularization in repairing ischemic injuries, we next examined the role of MIF in mediating the angiogenic function of ECs using tube formation and migration assays. As shown in Fig. 1C and D, OGD treatment significantly inhibited tube formation and cell migration compared to normoxic conditions. However, OGD-treated cells supplemented with either EPC-CM or rMIF exhibited a marked improvement in both tube formation and cell migration (Fig. 1C, D). These data indicate that both EPC-CM and rMIF enhance angiogenesis in an in vitro ischemic model.

### MIF is an important factor in EPC-CM that promotes angiogenesis in ischemic ECs

**Fig. 2:**
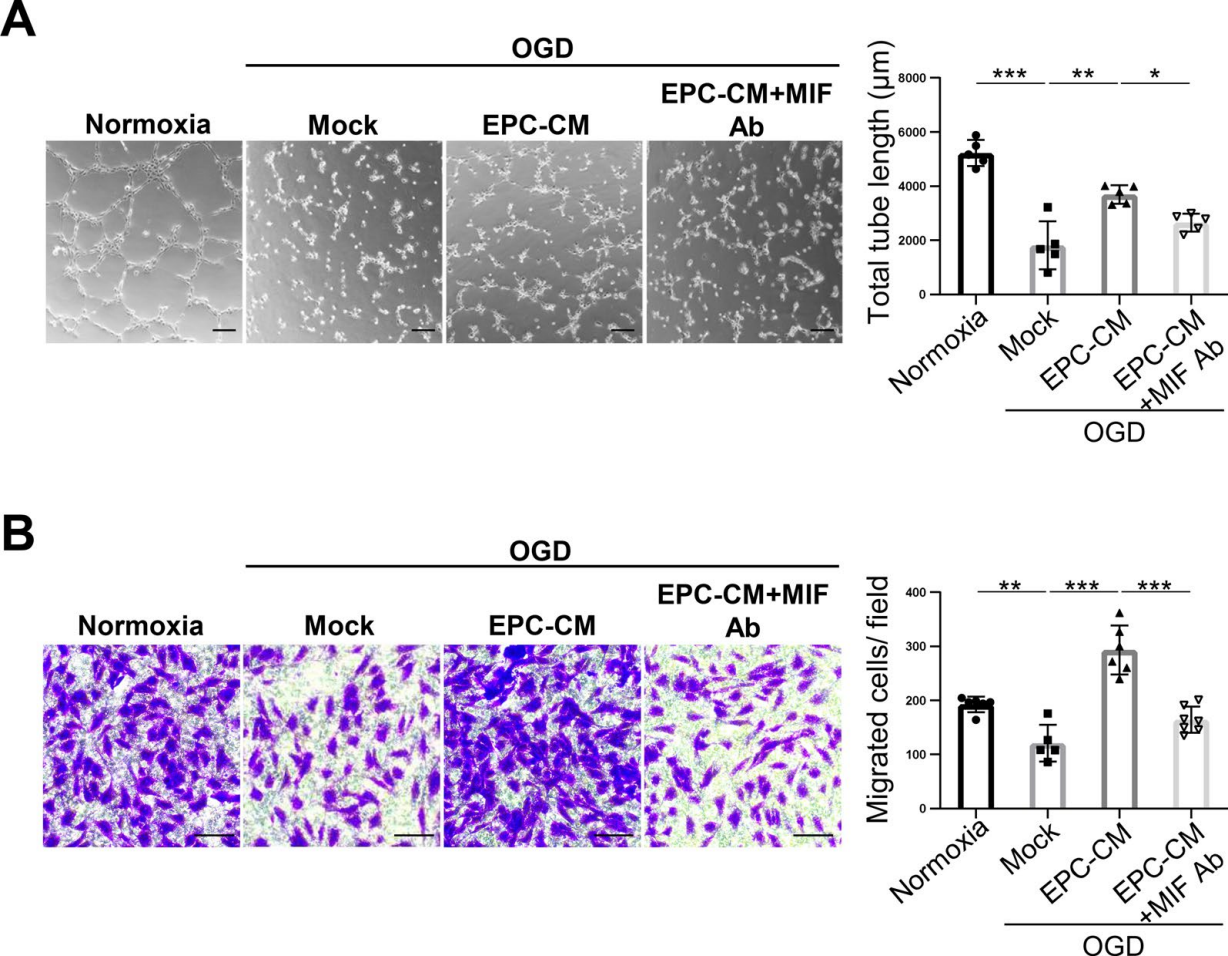
MIF Ab abolishes the enhancement of angiogenesis by EPC-CM in vitro. **A** Representative images of tube formation affected by each condition was examined by Matrigel assay (Normoxia: 20% basal medium, OGD/Mock: 20% basal medium, EPC-CM: 20% EPC-CM, MIF Ab: 20% basal medium with 1 µg/ml MIF Ab) (left panel). The average of total tube length in 5 random fields was quantitatively analyzed by MetaMorph software and shown as bar graph (right panel). Scale bar = 100 μm. **B** Determination of the HUVECs treated with different conditions described in (A) passed through the transwell membrane stained with the crystal violet (left panel). The migrated cell numbers were quantitatively analyzed by 6 fields randomly (right panel). Scale bar = 100 μm. The data was shown as the mean ± SD and analyzed by One-way ANOVA. * *p* < 0.05, ** *p* < 0.01, *** *p* < 0.001

To further validate the role of MIF in EPC-CM that plays in promoting the angiogenesis of ECs, we used anti-MIF antibody (MIF Ab) to specifically block the effect of MIF in EPC-CM. ECs were co-treated with EPC-CM and MIF Ab and maintained under OGD for 24 h, followed by angiogenic analysis. As shown in Fig. 2A and B, the significant improvement of tube formation and migration capacity observed in OGD-treated cells with EPC-CM were notably inhibited when co-treatment EPC-CM with MIF Ab (Fig. 2A, B, lane 3 vs. lane 4 in the bar graphs). These results strongly suggest that MIF is a key factor within EPC-CM responsible for promoting angiogenesis in the ischemic events.

### EPC-CM and MIF protein promote anti-senescence ability in EPCs and ECs

**Fig. 3:**
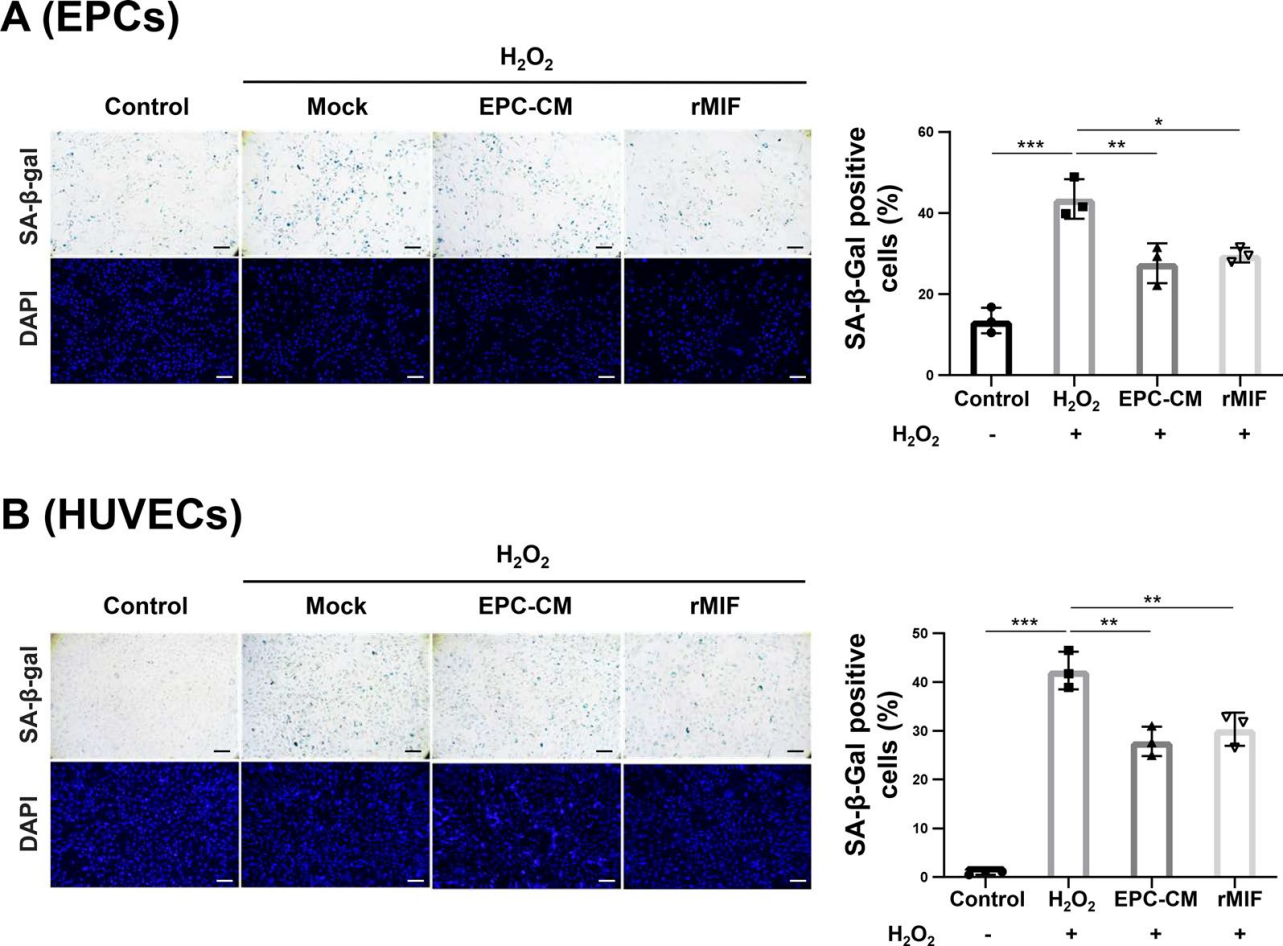
EPC-CM and MIF protein protect EPCs and ECs against the H_2_O_2_-induced senescence. **A** and **B** Representative micrographs of SA-β-gal staining and DAPI staining of H_2_O_2_-treated young EPCs (**A**) or the HUVECs (**B**) (passage < 10) that treated with/without EPC-CM or recombinant MIF protein (Control: 20% basal medium, H_2_O_2_/Mock: 20% basal medium, EPC-CM: 20% EPC-CM, rMIF: 20% basal medium with 100 ng/ml rMIF) (left panel). The senescence ratio was quantitatively analyzed of the SA-β-gal positive cells by 5 independent fields and normalized with DAPI staining. The data was compared with H_2_O_2_ group (right panel). Scale bar = 100 μm. The data was shown as the mean ± SD and analyzed by One-way ANOVA. * *p* < 0.05, ** *p* < 0.01, *** *p* < 0.001

Senescence is associated with neurodegenerative diseases [35]. Senescent EPCs showed decreased proliferation and angiogenesis. Therefore, we determined the anti-senescence effect mediated by EPC-CM or MIF on EPCs and ECs. Young EPCs or HUVECs underwent extended exposure to hydrogen peroxide (H_2_O_2_) to induce senescence, followed by treatment with EPC-CM (20%) or rMIF (100 ng/ml). H_2_O_2_ treatment significantly increased the number of senescent cells compared to the control (Fig. 3A, B, lane 1 vs. lane 2 in the bar graphs). Notably, EPC-CM or rMIF treatment reversed the senescence level in EPCs (EPC-CM, 27.67% ± 4.01; rMIF, 29.67% ± 1.47 vs. H_2_O_2,_ 43.48% ± 3.95; both *p* < 0.05; Fig. 3A) and HUVECs (EPC-CM, 27.82% ± 2.46; rMIF, 30.34% ± 2.75 vs. H_2_O_2,_ 42.37% ± 3.14;* p* < 0.01 and *p* < 0.05, respectively; Fig. 3B). These data indicated that EPC-CM and MIF may promote angiogenesis by rejuvenating senescent EPCs or ECs after ischemic damage.

### Transduction of EPCs with lenti-hMIF promotes the angiogenesis and anti-senescence in injured cells

To further validate the role of MIF in promoting angiogenesis and preventing senescence, we generated human MIF (hMIF)-expressing EPCs using a lentivirus and confirmed hMIF expression through Western blot analysis (*p* < 0.001; Additional file 1: Fig. S3A). The angiogenesis and anti-senescence capacity were tested in the hMIF-expressing EPCs compared with lentiviral vector-transduced EPCs (Vector). As shown in Additional file 1: Fig. S3B and S3C, hMIF-expressing EPCs rescued the tube formation and migration repressed by OGD treatment as compared with OGD-treated EPCs transduced with lentiviral vector (*p* < 0.001). In addition, hMIF-expressing EPCs also restored the senescence level after H_2_O_2_ induction (*p* < 0.001; Additional file 1: Fig. S3D), consistent with the findings in Fig. 3. These data highly support MIF is an important protein that regulates angiogenesis and anti-senescence in EPCs.

### Treatment with MIF Ab abolishes the vascular repair mediated by EPC-CM in BICAL rats

**Fig. 4:**
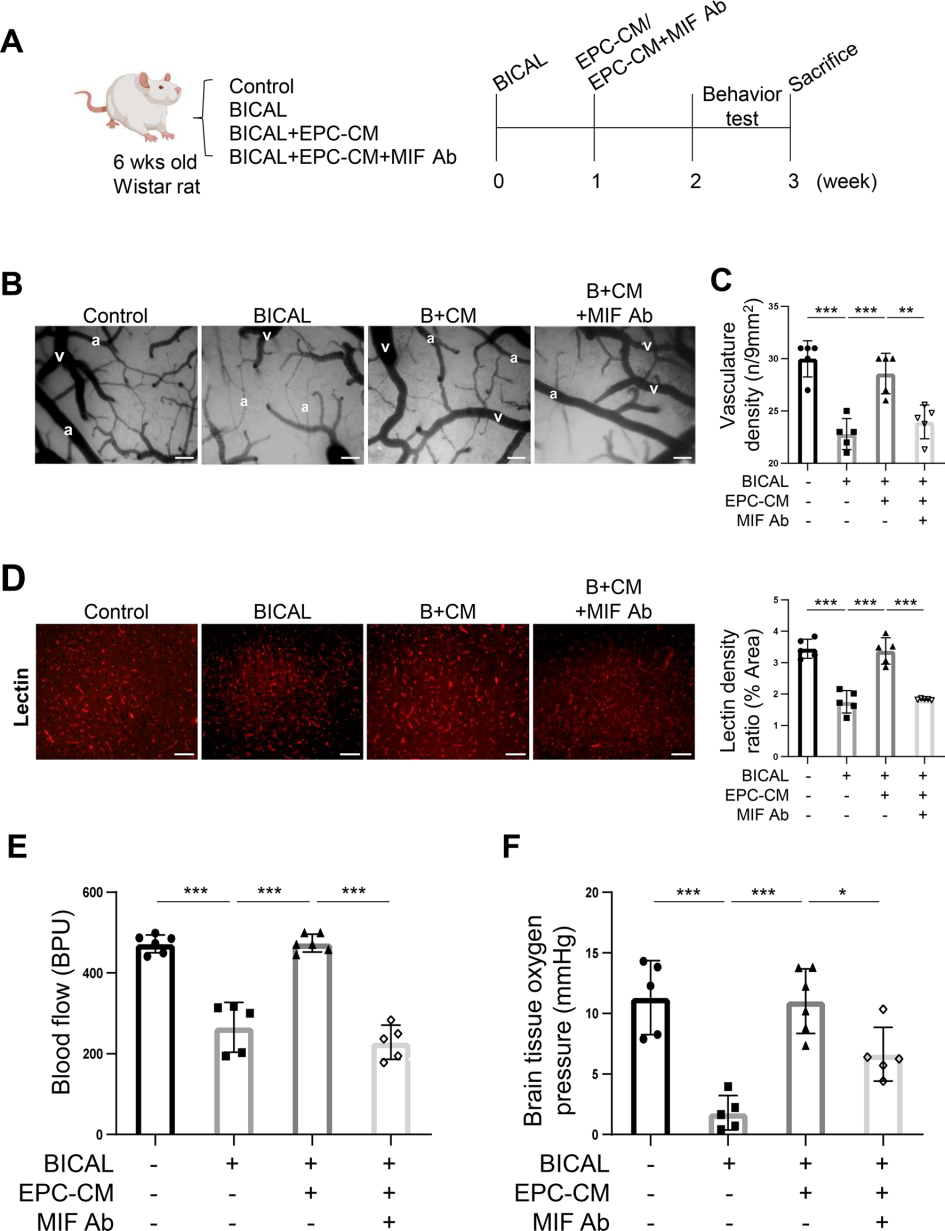
MIF is the key factor in the EPC-CM to promote the vascular repair after BICAL. **A** The schematic representation of the experimental process. **B** The representative micrographs of microcirculation on the brain surface recorded by the videoscope (arteriole marketed as “**a**” and vein labeled as “**v**”; B + CM: BICAL + EPC-CM). Scale bar = 50 µm. **C** The effects of EPC-CM and MIF Ab on the cerebral microvasculature density of BICAL rats were quantified by using a CAM1 capillary anemometer (n = 5). **D** Representative images of immunostaining of LE-lectin in cortex of different treatments of Wistar rats (left panel, B + CM: BICAL + EPC-CM). Scale bar = 50 μm. The density of LE-lectin was quantified by using ImageJ (right panel). Each symbol represents the average density of 5 independent cortical fields per rat (n = 5). **E** and **F** On BICAL rats, the vascular repair effects of EPC-CM and MIF Ab were evaluated by regional blood flow (**E**) and the partial pressure of brain tissue oxygen (PbtO_2_) determination (n = 5) (**F**). The data are presented as mean ± SD, and analyzed using One-way ANOVA. * *p* < 0.05, ** *p* < 0.01, *** *p* < 0.001

Previous in vitro data suggest EPC-CM emerges as a promising cell-free therapeutic approach for ischemic events, with MIF playing a critical role in mediating angiogenesis. To further investigate the therapeutic effects of EPC-CM and the potential role of MIF in the treatment of CCI, BICAL was conducted as the CCI animal model. The EPC-CM with/without MIF Ab was injected into the cisterna magna of the experimental rats one week after BICAL surgery. The vascular system and behavior tests were performed three weeks post-BICAL to evaluate the impact of the EPC-CM or MIF Ab on the vascular and neuronal repair in BICAL rats (Fig. 4A). The microcirculation on the cortical surface, including arterioles and veins, was significantly sparser in BICAL rats compared to controls, with arterioles exhibiting pronounced diffuse vasoconstriction (Fig. 4B). The vasculature density decreased notably in BICAL rats compared to the control group (Fig. 4C). Additionally, the cortical blood vessels in BICAL rats labeled with tomato LE-lectin (LEL), a widely used endothelial marker, exhibited lower vessel density than the control, reflecting the changes observed in cortical surface microcirculation (Fig. 4D). Furthermore, the regional blood flow, showed as blood perfusion units (BPU), and partial pressure of brain tissue oxygen (PbtO_2_) also declined significantly in the BICAL rats, indicating a state of hypoperfusion (Fig. 4E, F, lane 2 vs. lane 1 in the bar graphs). Notably, the impairments of microvasculature, blood flow, and PbtO_2_ in BICAL rats were effectively reversed by the treatment of EPC-CM, whereas co-treatment with MIF Ab diminished these improvements (Fig. 4B-F). These data suggest that EPC-CM improved vascular repair and cerebral perfusion after BICAL through MIF-mediated regulation.

### MIF is an important factor in EPC-CM that promotes neurologic functional recovery in BICAL rats

**Fig. 5:**
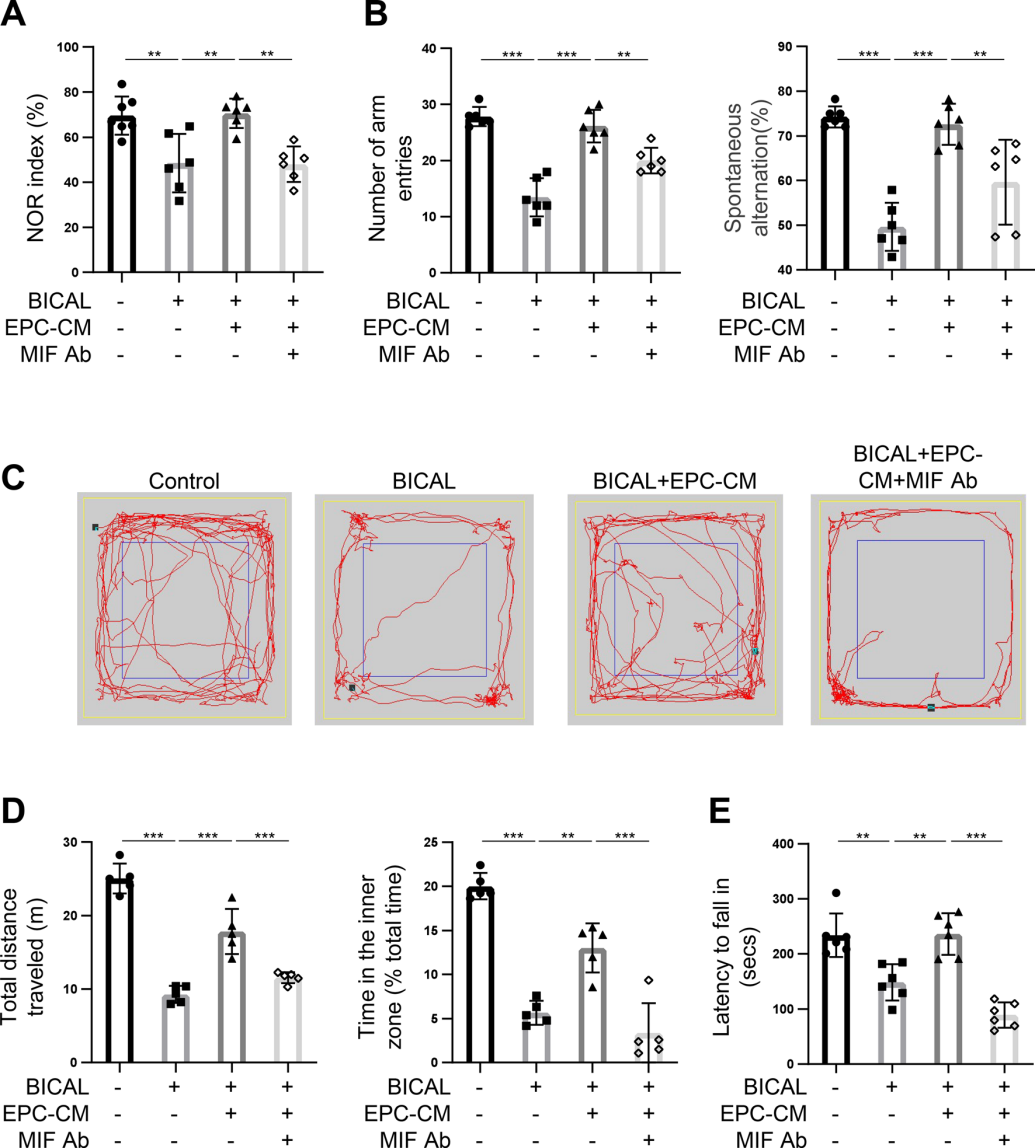
MIF plays an important role in EPC-CM-mediated repair of the cognitive and motor impairments after BICAL. **A** The memory function of BICAL rats was evaluated by the NOR test. The duration of time spent exploring the novel object was normalized with total time spent exploring both objects (n = 6). **B** The spatial working memory of BICAL rats was evaluated by Y-maze, including the numbers of total arm entries (left panel) and the percentage of spontaneous alternation (%) (right panel) (n = 6).** C** Representative track plots showed the paths of control and BICAL rats treated with/without EPC-CM or MIF Ab in the OFT over a 5 min duration. **D** The total distance traveled indicated the locomotor activity of each group (left panel). The anxiety-related behaviors were evaluated by the time spent in the center area (% of total time, right panel) in each group (n = 5). **E** The effects of EPC-CM or EPC-CM + MIF Ab treatment on the motor function after BICAL were evaluated by rotarod test (n = 6). The data was shown as the mean ± SD and analyzed by One-way ANOVA. * *p* < 0.05, ** *p* < 0.01, *** *p* < 0.001

The CCI correlates to functional neurological disorder, especially the cognitive impairment [36]. To further explore the role of MIF within EPC-CM to neuronal repair in BICAL rats, we conducted a series of behavioral assessments, including cognition, locomotor activity and anxiety in the BICAL rats. These behaviors are known to be related to the neurological function in experimental animals. Cognitive function was assessed through the novel object recognition (NOR) assay to test the short-term memory, and the Y-maze test for the spatial working memory. Motor function was evaluated using the rotarod test. The locomotor and anxiety-related behaviors were evaluated by the open field test (OFT). In the NOR test (Fig. 5A) and Y-maze test (Fig. 5B), the BICAL rats showed fewer exploration time and entry numbers in the novel subject and novel arms than the control, whereas EPC-CM treatment significantly rescued the decline (Fig. 5A,B, lane 2 vs. lane 3 in the bar graphs). This suggested EPC-CM administration repaired the decline in recognition and spatial memory caused by CCI. Additionally, by comparing the travel paths and thigmotaxis in the OFT (Fig. 5C,D) and the rotarod data (Fig. 5E), EPC-CM treated rats had improvement of the motor function, the locomotor impairments, and anxiety induced by BICAL. Consistent with earlier findings, co-treatment with MIF Ab negated the beneficial effects of EPC-CM on the cognitive and motor impairments in BICAL rats (Fig. 5A-E, lane 3 vs. lane 4 in the bar graphs). Taken together, these results strongly support that MIF is the key factor in EPC-CM responsible for mitigating vascular and neurological damages associated with CCI.

### MIF promotes angiogenesis and anti-senescence by activating the AKT pathway

**Fig. 6:**
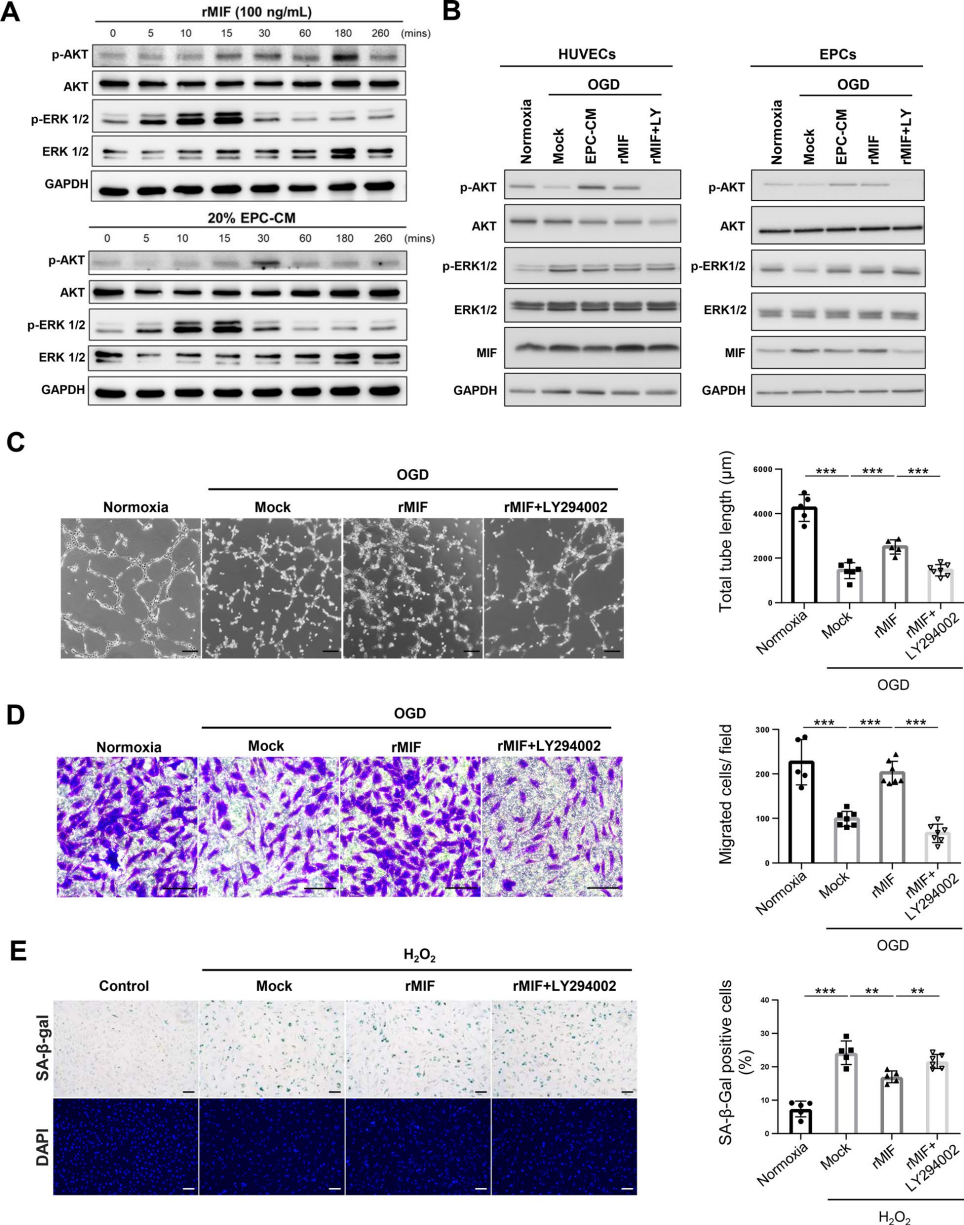
MIF promotes the angiogenesis and anti-senescence via activating the AKT pathway. **A** Determination the AKT and ERK1/2 activity upon the treatment of EPC-CM or recombinant MIF protein in different time points in the EPCs. The EPCs were treated with rMIF (100 ng/ml) or EPC-CM (20%) for the indicated time and harvested the cell lysates for Western blotting analysis. Full-length blots/gels are presented in Additional file 1: Fig. S4. **B** Detection of the AKT and ERK1/2 activity in OGD-treated cells incubated with EPC-CM, or rMIF, or rMIF + LY (LY294002, 5 µM). 90% confluency HUVECs (left panel) or EPCs (right panel) were incubated with 5% FBS EGM-2 with/without EPC-CM (20%), or rMIF (100 ng/ml), or the rMIF + LY294002 (5 µM) at 1% O_2_ incubator for 24 h. The cell lysates were collected to detect the AKT and ERK1/2 activity by using the Western analysis. Full-length blots/gels are presented in Additional file 1: Fig. S5. **C** Representative images of the tube formation of the OGD-treated cells incubated with different chemicals mentioned in (B) (left panel). The average total tube length of five random fields was quantified and showed as bar graph (right panel). Scale bar = 100 μm.** D** Representative images of the HUVECs passed through the transwell membrane stained with crystal violet (left panel). The migrated cell numbers were quantitative analyzed by 6 fields randomly (right panel). Scale bar = 100 μm. **E** Images of the SA-β-gal staining of H_2_O_2_-induced senescent cells treated with different chemicals in HUVEC cells. Young HUVECs (passage < 10) treated H_2_O_2_ (200 µM) for two days, followed with rMIF, or rMIF + LY294002 (5 µM) treatment for three days (left panel). The senescence ratio was the SA-β-gal positive cells normalized with the DAPI in 6 independent fields. The data were compared with the H_2_O_2_ group (right panel). Scale bar = 100 μm. The data was showed as the mean ± SD, and analyzed using One-way ANOVA. ** *p* < 0.01, *** *p* < 0.001

Having established the role of MIF in promoting angiogenesis in a CCI model of rats, we investigated the underlying mechanism. Previous studies demonstrated that MIF increased angiogenesis and anti-senescence by mediating the phosphatidylinositol 3'-kinase (PI3K) /AKT signaling pathway and mitogen-activated protein kinase (MAPK) pathway [32, 37]. Therefore, we investigated the effect of MIF or EPC-CM on ERK1/2 and AKT activity in EPCs. EPCs treated with rMIF or EPC-CM both significantly increases the ERK1/2 and AKT phosphorylation, consistent with previous studies (Fig. 6A). Next, we detected the ERK1/2 and AKT activity of ECs and EPCs under OGD condition with/without EPC-CM or rMIF treatment. The AKT activity was inhibited by OGD in ECs and EPCs, which was recovered by treatment with EPC-CM or rMIF treatment. However, the ERK1/2 activity was unaffected by the treatment of EPC-CM or rMIF in the ECs (Fig. 6B). The data suggested that AKT is the critical pathway regulated by MIF in ECs and EPCs to promote angiogenesis and anti-senescence in ischemic condition. To validate this hypothesis, we used a PI3K/AKT inhibitor (LY294002) to block the AKT activity, and the inhibitory efficacy was assessed by Western blotting (Fig. 6B, lane 5). As shown in Fig. 6C and D, the tube formation and cell migration promoted by rMIF treatment under OGD condition were abolished by the co-treatment of LY294002 (*p* < 0.001). In addition, the anti-senescence effect mediated by rMIF was repressed by LY294002 (*p* < 0.01; Fig. 6E). Taken together, these data suggested EPC-CM promoted angiogenesis and anti-senescence to mitigate chronic ischemic injury through MIF-activated AKT pathway.

## Discussion

Chronic cerebral ischemia is a global health concern that is closely associated with neurodegenerative diseases, contributing to increased mortality and disability rates in aging populations. Early treatment of CCI is crucial for preventing vascular dysfunction and mitigating the risk of subsequent pathological events, including stroke, dementia, and AD. Unfortunately, effective therapies for CCI remain limited. In this study, we identified MIF, a pleiotropic cytokine, as a critical factor in EPC-CM that exhibits important angiogenic activity and anti-senescence activity, facilitating cells recovery in vitro. We further demonstrated EPC-CM treatment mitigated the impairment of microcirculation, cognition, and motor function of the CCI animals. Additionally, we found out that EPC-CM promoted angiogenesis and anti-senescence in EPCs and ECs through MIF-mediated AKT activation. Collectively, this study underscores the therapeutic potential of EPC-CM in CCI.

Vascular repair is critical for CCI. EPCs, as the precursor cells of ECs, play a pivotal role in repairing injured vessels and participate in endothelial regeneration [8, 38]. Despite their attractiveness as a therapeutic approach for ischemic diseases, challenges such as immunogenicity, tumorigenic potential, and formation of emboli limit the clinical application of EPCs transplantation [17]. The detailed mechanism by which EPCs contribute to vascular repair is still unclear. There are two mechanisms by which EPCs promote vascular repair: one is that EPCs migrate to the ischemic region and differentiate into ECs to repair damaged vessels, and the other is that the recruited EPCs secrete protective paracrine factors to promote vascular repair [39]. The latter mechanism, particularly relevant for its stability, safety, and low immunogenicity, has gained prominence. Evidence supports that EPC-mediated vascular repair via paracrine signaling to activate resident ECs is more critical than direct differentiation into damaged vessels [18, 40, 41]. In the current study, we also demonstrated that the administration of EPC-CM facilitates cerebral vascular and neuronal repair after ligation of bilateral ICAs in rats. The angiogenesis and anti-senesces effect of EPC-CM may repair the damaged vessels in the ischemic regions caused by CCI. In addition, the paracrine factors in EPC-CM may modulate the downstream injurious processes that include oxidative stress, neuroinflammation, and apoptosis as a consequence of CCI. The results indicate the EPC-CM may provide therapeutic effects on CCI by reducing the ischemic injury and attenuating the subsequent downstream damages.

The cytokine profile of EPC-CM highlighted MIF as a key factor in promoting angiogenesis and cell survival. The validation of the regulation mechanism in in vitro system revealed that MIF and EPC-CM increase angiogenesis and anti-senescence capacity in OGD or H_2_O_2_ treated cells (Figs. 1 and 3). The role of MIF in promoting of angiogenesis and anti-senescence were also validated by MIF Ab treatment and lenti-MIF expressing EPCs in vitro (Fig. 2 and Additional file 1: Fig. S3). These data supported the therapeutic effects of EPC-CM and MIF in ischemic condition.

Furthermore, BICAL, a CCI animal model was conducted to investigate the effects of EPC-CM and MIF on the ischemic brains. The microcirculation changes including the decrease of vascular density, regional blood flow, and PbtO_2_ and the impaired motor and cognitive functions after BICAL were effectively ameliorated by the administration of EPC-CM. And the improvements mediated by EPC-CM in BICAL rats were significantly abolished when co-treatment with MIF Ab (Figs. 4 and 5). The in vivo findings confirmed the potential role of EPC-CM in the management of CCI through the regulation of MIF.

MIF was first identified as an inflammatory cytokine and is now recognized as a protective factor that involves in diverse physiological processes, including angiogenesis, antioxidant activity, and cell survival [23, 42]. Many studies have demonstrated that MIF participates in the recovery of ischemic injury [43–46]. However, some studies reported that MIF aggravated the ischemic damage [47, 48]. Inácio et al. found a smaller infarct size 7 days after middle cerebral artery occlusion (MCAO) in MIF-knockout mice [48]. These studies pointed out that MIF has different roles during ischemia, either promoting neuronal recovery by inhibiting cell death or being harmful to neurons by increasing the inflammatory response in ischemic region, and the mechanism underlying the different roles of MIF is still unclear. One possibility is that high expression of MIF may activate broad signaling pathways and inflammation. In the rodent permanent MCAO model, MIF showed detrimental effects, which increased the permeability of blood brain barrier and the infarction volume with 3.3 µg/kg administration in Liu’s study [49], whereas 0.9 ng MIF injected into mice via the ventricle (final 120 ng/ml concentration) promoted neurological recovery in the rodent MCAO model [43]. The MIF concentration in EPC-CM was 12 ng/ml, and treatment with 40 µl EPC-CM in BICAL rats showed significant protective effects in our study, consistent with the low dose of MIF administered in the stroke model [43].

Secreted MIF activates multiple cellular signaling pathways by binding to the cellular receptor CD74 [50], which triggers various signaling pathways, including the MAPK and PI3K/AKT pathways, which are crucial for angiogenesis and preventing senescence [32, 37]. In addition, MIF can bind to CXCR2 and CXCR4 receptors to regulate cell proliferation, survival, angiogenesis, and chemotaxis [51, 52]. Here, we demonstrated that MIF promotes angiogenesis via activation of the PI3K/AKT pathway to increase angiogenesis and anti-senescence of EPCs and ECs (Fig. 6). The regulation of MIF on the PI3K/AKT pathway was validated using the PI3K inhibitor LY294002, which significantly reduced AKT phosphorylation and inhibited the associated improvements in angiogenesis and anti-senescence in EPCs and ECs (Fig. 6). However, LY294002 affects not only the AKT pathway but also other PI3K downstream signaling processes, such as macropinocytosis [53]. Therefore, the regulation of MIF on PI3K downstream signal pathways could not be differentiated in this study. Future studies using more specific inhibitors or genetic approaches to specifically target AKT or macropinocytosis would help clarify the precise mechanisms underlying MIF-mediated effects.

Cerebral small vessel impairment is related to various diseases and the treatment is crucial for the prevention of the neurodegenerative diseases like dementia [54]. This study proved the therapeutic effect of EPC-CM in animals with CCI. Notably, our study differed from prior researches where CM was administered for multiple times after stroke [13, 15]; here, EPC-CM was delivered once, 7 days following BICAL surgery, yet still exhibited a substantial vascular repair effect. This indicates that the EPC-CM used in our study had long-term effects on EPC-mediated re-endothelialization and neovascularization, which might be due to EPCs chemotaxis to the ischemic region mediated by MIF [21, 55]. An intriguing avenue for exploration is whether the vascular repair facilitated by EPC-CM could extend to various ischemic diseases. Further investigation is needed to elucidate the detailed mechanism of EPC-CM and MIF after ischemic events. This includes investigating the chemotactic response mediated by MIF and optimizing the MIF dosage in EPC-CM for its application in ischemic diseases.

## Conclusion

In conclusion, our study highlighted the therapeutic potential of EPC-CM for vascular and neuronal recovery in CCI. The pivotal role of MIF in angiogenesis and anti-senescence through the activation of the PI3K/AKT pathway provides mechanistic insights into the neuroprotective effects of EPC-CM. These findings hold promise for developing novel therapies for cerebral ischemic diseases.

## Supplementary Information

**Additional file 1.**

## Data Availability

The data that support the findings of this study are available from the corresponding authors upon reasonable request.

## Abbreviations

CCI: Chronic cerebral ischemia
BICAL: Bilateral internal carotid artery ligation
EPCs: Endothelial progenitor cells
EPC-CM: Endothelial progenitor cell-derived conditioned medium
MIF: Macrophage migration inhibitory factor
OGD: Oxygen–glucose deprivation
ECs: Endothelial cells
HUVEC: Human umbilical vein endothelial cell
SA-β-gal: Senescence associated β-galactosidase
OFT: Open field test
NOR: Novel object recognition
