# Supplementary material for: Endothelial progenitor cell-derived conditioned medium mitigates chronic cerebral ischemic injury through macrophage migration inhibitory factor-activated AKT pathway
Source: Stem Cell Res Ther. 2024 Nov 14;15:428. doi: 10.1186/s13287-024-04015-5 (PMC11566597; doi:10.1186/s13287-024-04015-5)
Supplement: Supplementary file 1 — Additional file 1. [file 13287_2024_4015_MOESM1_ESM.pdf]

Endothelial progenitor cell-derived conditioned medium mitigates chronic cerebral ischemic injury through macrophage migration inhibitory factor-activated AKT pathway

Ya-Wen Cheng[1], Ling-Yu Yang[1], Yi-Tzu Chen[1,2], Sheng-Che Chou[1,2], Kuo-Wei Chen[1,2], Yi-Hsing Chen[1,2], Chuan-Rou Deng[1], I-Chin Chen[1], Wan-Ju Chou[1,2], Chen-Chih Chang[3], Yong-Ren Chen[4,5], Hsiao-Lin Hwa[6], Kuo-Chuan Wang[1*], and Meng-Fai Kuo[1*]

Short title: **The role of MIF in chronic cerebral ischemia**

Affiliations of authors:

1 Division of Neurosurgery, Department of Surgery, National Taiwan University Hospital, Taipei, Taiwan

2 Graduate Institute of Clinical Medicine, National Taiwan University College of Medicine, Taipei, Taiwan

3 Department of Internal Medicine, National Taiwan University Hospital, College of Medicine, National Taiwan University, Taipei, Taiwan

4 Non-invasive Cancer Therapy Research Institute, Taipei, Taiwan

5 Adjunct Visiting Staff, Division of Neurosurgery, Department of Surgery, National Taiwan University Hospital, Taipei, Taiwan.

6 Department of Obstetrics and Gynecology, National Taiwan University Hospital, Taipei, Taiwan

[*]Corresponding authors: Meng-Fai Kuo, mfkenator@gmail.com and Kuo-Chuan Wang, wang081466@yahoo.com.tw

Table S1 The human cytokine array results from conditioned medium of EPCs

	Protein name	EPC-CM (<5%)	EPC-CM (5-30%)		Protein name	EPC-CM (<5%)	EPC-CM (5-30%)		Protein name	EPC-CM (<5%)	EPC-CM (5-30%)
1	PAI-1	3.65	18,637.18	47	DKK-1	0.52	25.10	93	Dtk	6.80	0.93
2	ICAM-2	71.66	10,729.11	48	TREM-1	16.35	24.68	94	EGF R	0.00	0.91
3	MIF	13.33	8,018.72	49	Galectin-7	72.36	22.35	95	MIP-1d	0.00	0.80
4	IGFBP-4	641.66	2,295.08	50	DR6	0.50	21.91	96	EG-VEGF	3.81	0.78
5	Angiostatin	1,887.46	1,914.17	51	IL-2 Ra	34.12	20.28	97	bFGF	4.68	0.73
6	TIMP-2	2.28	1,421.55	52	I-TAC	22.97	20.22	98	BTC	5.22	0.67
7	TIMP-1	0.00	1,317.97	53	XEDAR	5.10	20.04	99	VEGF	0.00	0.65
8	IL-13 R2	244.11	979.02	54	Eotaxin	2.15	16.03	100	TARC	0.56	0.46
9	VEGF R1	0.00	728.14	55	TNF RII	3.05	14.28	101	NRG1-b1	2.98	0.32
10	TRAIL R3	4.04	539.34	56	IL-15	32.48	13.22	102	NT-4	3.59	0.29
11	IGFBP-2	3.58	533.83	57	SCF R	0.00	12.54	103	MIG	0.31	0.24
12	MCP-1	5.82	481.05	58	Cathepsin S	5.82	10.53	104	BDNF	0.14	0.21
13	HCC-1	1.50	325.24	59	IL-6	4.19	10.42	105	IL-12p70	0.32	0.18
14	IL-17B	76.01	239.46	60	Shh-N	0.00	9.75	106	BLC	0.04	0.14
15	DAN	0.00	236.07	61	LIMPII	0.30	9.41	107	MIP-1b	0.93	0.13
16	ICAM-1	0.32	177.37	62	IL-17R	18.36	8.98	108	MIP-3a	0.15	0.12
17	GDF-15	0.20	156.55	63	CEACAM-1	6.21	8.86	109	IL-11	7.30	0.11
18	PIGF	0.04	134.45	64	IL-6R	2.61	8.57	110	GCP-2	0.00	0.10
19	IL-13 R1	303.09	124.85	65	Endoglin	1.78	8.16	111	MCP-3	0.10	0.02
20	PECAM-1	9.32	110.14	66	TSLP	10.27	7.38	112	IL-9	17.60	0.00
21	E-Selectin	0.04	78.41	67	CXCL16	0.00	7.36	113	IL-18 BPa	2.73	0.00
22	OPG	0.00	71.39	68	ST2	6.17	7.14	114	IL-31	0.47	0.00
23	gp130	0.00	71.21	69	MICA	4.42	7.09	115	LIGHT	1.04	0.00
24	Activin A	253.30	67.88	70	ErbB3	1.37	4.65	116	MCP-4	0.16	0.00
25	TGFb1	170.68	67.13	71	Cripto-1	2.69	4.56	117	MIP-3b	0.00	0.00
26	E-Cadherin	34.58	66.18	72	MCSF	0.24	4.51	118	MSP	13.89	0.00
27	Follistatin	8.43	64.12	73	L-Selectin	47.30	4.04	119	PARC	2.55	0.00
28	LYVE-1	2.96	63.13	74	IL-2 Rg	12.83	4.00	120	SDF-1a	0.37	0.00
29	IL-13	68.25	62.40	75	BMP-4	4.35	3.63	121	BMP-5	307.48	0.00
30	IL-8	2.09	61.65	76	TGFb2	2.54	3.63	122	GH	3.71	0.00
31	LAP(TGFb1)	3.06	57.85	77	GRO	1.44	3.49	123	VEGF-D	0.15	0.00
32	VCAM-1	97.70	55.79	78	IGFBP-1	5.00	3.47	124	GM-CSF	2.70	0.00
33	ANG-1	64.41	50.56	79	ALCAM	0.00	2.64	125	I-309	1.66	0.00
34	uPAR	36.23	47.88	80	IL-1a	1.96	2.21	126	IL-1b	0.02	0.00
35	TNF RI	2.88	45.80	81	IL-1ra	0.00	2.16	127	IL-7	0.86	0.00
36	TNFa	33.03	42.78	82	HGF	0.00	1.75	128	IL-10	0.79	0.00
37	PDGF-AB	27.77	40.14	83	OPN	2.60	1.69	129	IL-12p40	0.24	0.00
38	Tie-2	28.63	39.03	84	IL-17	0.00	1.68	130	IL-16	0.49	0.00
39	AR	25.25	38.99	85	RANTES	0.57	1.64	131	TNFb	16.04	0.00
40	IL-2 Rb	52.72	37.19	86	Lipocalin-2	1.97	1.52	132	CD40L	6.44	0.00
41	Eotaxin-3	43.81	35.89	87	IP-10	1.22	1.48	133	ICAM-3	4.32	0.00
42	NrCAM	12.12	35.22	88	VEGF R2	0.00	1.46	134	MICB	17.11	0.00
43	AgRP	38.27	31.93	89	IFNg	0.00	1.34	135	PDGF Rb	82.19	0.00
44	Axl	0.00	30.30	90	RAGE	2.31	1.22	136	TIM-1	12.39	0.00
45	Angiogenin	32.72	25.96	91	BCMA	6.29	1.08	137	Trappin-2	2.63	0.00
46	TRAIL R4	58.60	25.14	92	IL-2	0.59	1.03				

The medium was collected from young EPCs and filtered by a Tangential Flow Filtration (TFF) membrane filter system unit with a 30 kDa cut-off (Millipore). The filtered and

concentrated medium was studies with the cytokine array by using Quantitative Cytokine Quantibody Human Array 4000 (RayBiotech, Norcross, GA) following the manufacturer's protocol.

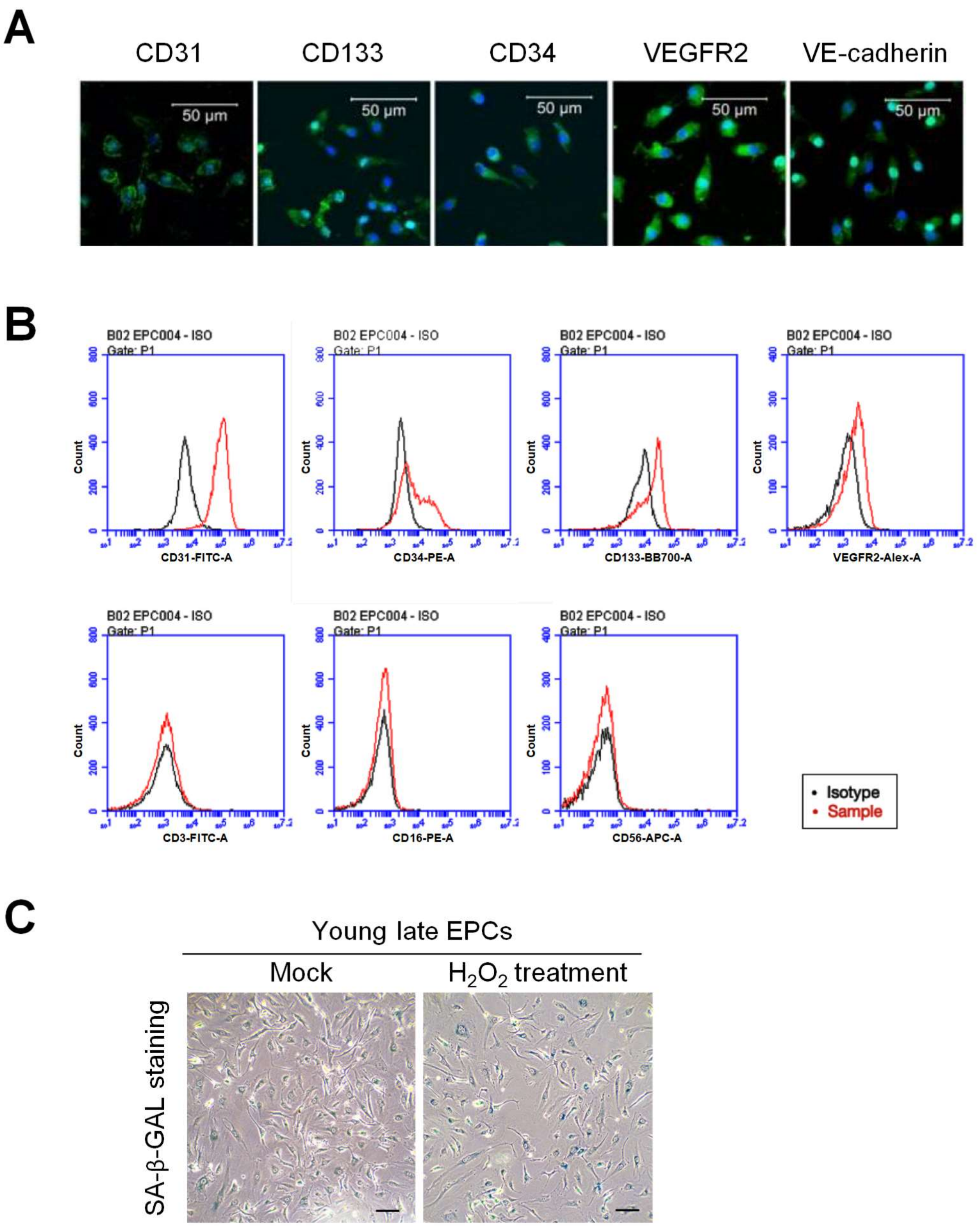

Fig. S1 Characterization of the isolated EPCs.

A Isolated EPCs express endothelial and hematopoietic stem cell markers, including CD133, CD31, CD34, VEGFR2, and VE-cadherin. Cells were counterstained with DAPI for the nuclei (blue). Scale bar = 50 µm. **B** The critical markers of EPC (CD34, CD133, CD31, VEGFR2) of the isolated EPCs were examined by flow cytometry, and the isotype Ab was used as negative control. The analysis showed the cells present EPC specific

markers (CD31+, CD34+, CD133+, VEGFR2+), but negative of general lymphocyte cell marker, CD3, CD16, and CD56. **C** The senescent status of young EPCs was detected by SA-β-GAL staining. The cells treated with H_2O_2 (200 μm) to induce cellular senescence were used as positive control. Scale bar = 100 μm.

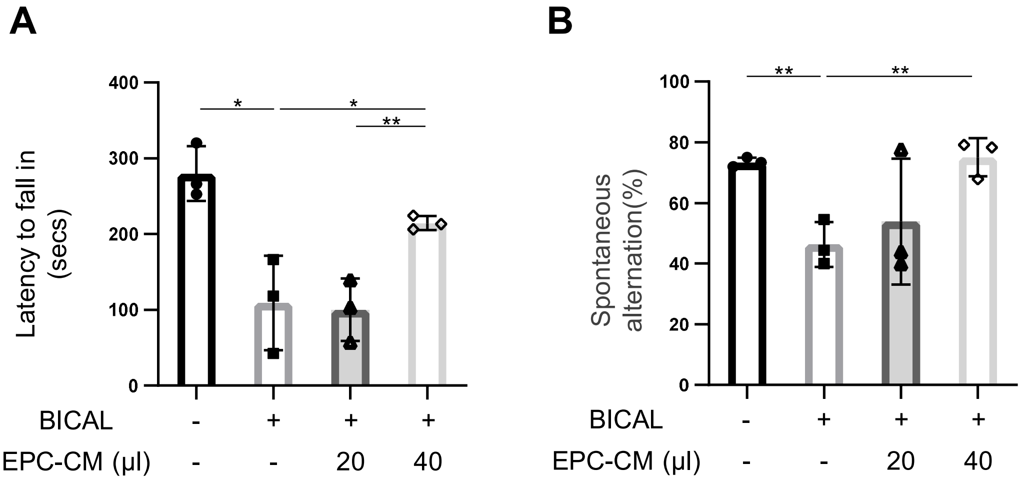

Fig. S2 Effect of different dosages of EPC-CM on the motor and cognitive functions in BICAL rats.

The dosage effect of EPC-CM treatment (20 or 40 μl) on the BICAL rats was evaluated. **A** The motor function was evaluated by rotarod test (n = 3). **B** The cognitive function was examined by Y-maze test (n = 3). The data was showed as the mean ± SD, and analyzed using Student *t* test. * $p < 0.05$, ** $p < 0.01$.

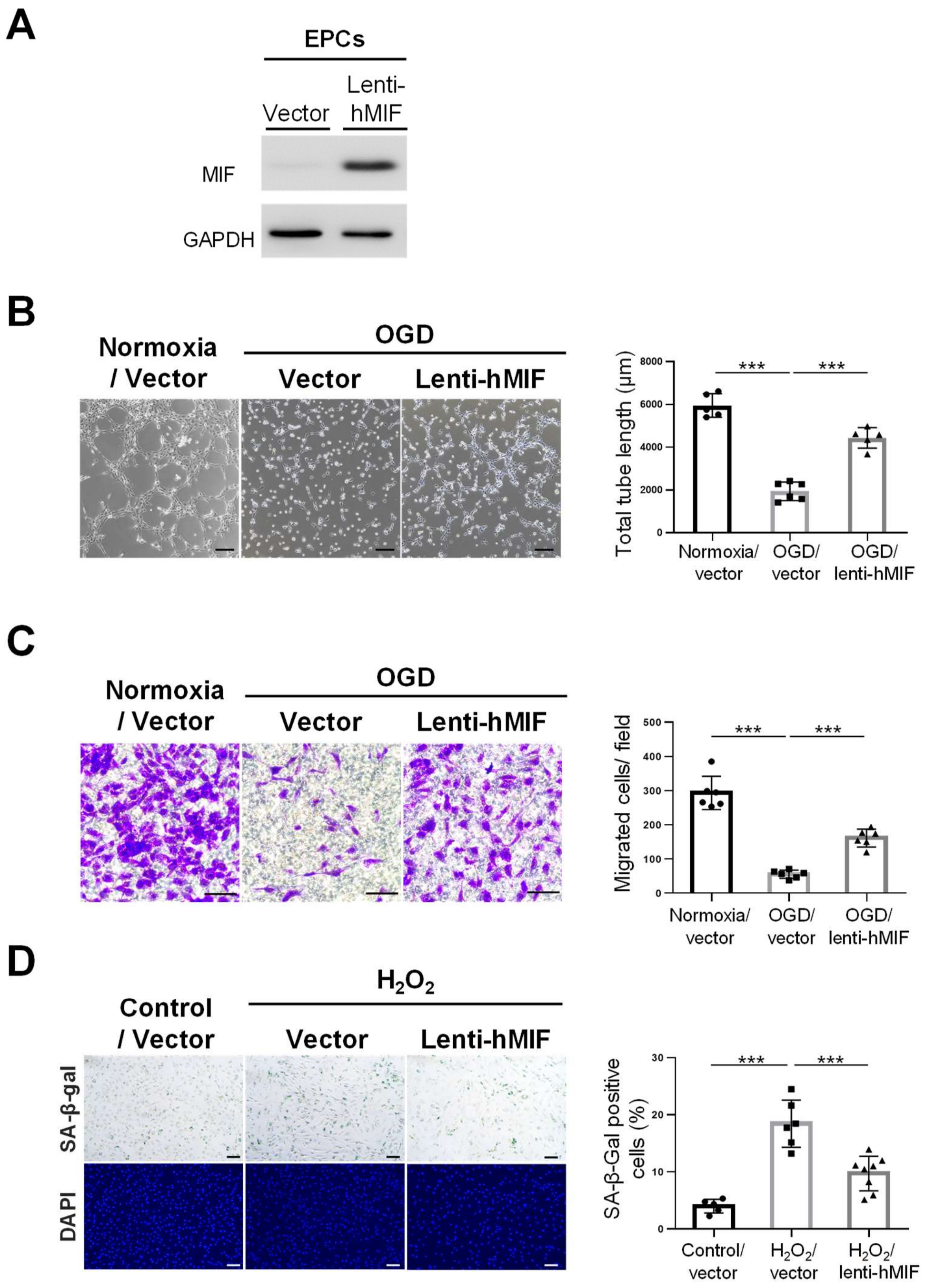

Fig. S3 Transduction of EPCs with lenti-hMIF increases cells angiogenesis and anti-senescence *in vitro*

A Detection of the MIF protein expression of the lenti-hMIF virus transduced cells. EPCs were transduced with lenti-hMIF virus for three days. The MIF expression was analyzed

by Western blotting using anti-MIF Ab. The lenti-vector transduced cells were used as control. **B** Determination of the tube formation by Matrigel assay in EPCs expressing hMIF with OGD treatment (left panel). The average total tube length in 5 random fields was quantitatively analysis and showed as bar graph (right panel). Scale bar = 100 μm. **C** Images of the EPCs passed through the transwell membrane stained by crystal violet staining (left panel). The migrated cell numbers were quantitatively analyzed by 6 fields randomly (right panel). Scale bar = 100 μm. **D** Images of the SA-β-gal staining in H_2O_2 treated EPCs. Young EPCs (passage < 10) was transduced with lenti-hMIF or lenti-vector control for 3 days. The transduced EPCs were treated with H_2O_2 to induce the senescence, then followed by 5% FBS EGM-2 incubation for three days (left panel). The senescence ratio was analyzed by the number of the SA-β-gal positive cells, and normalized with DAPI staining. The data were comparted with the H_2O_2 group (right panel). Scale bar = 100 μm. The data were showed as the mean ± SD, and analyzed using One-way ANOVA. ** $p < 0.01$, *** $p < 0.001$.

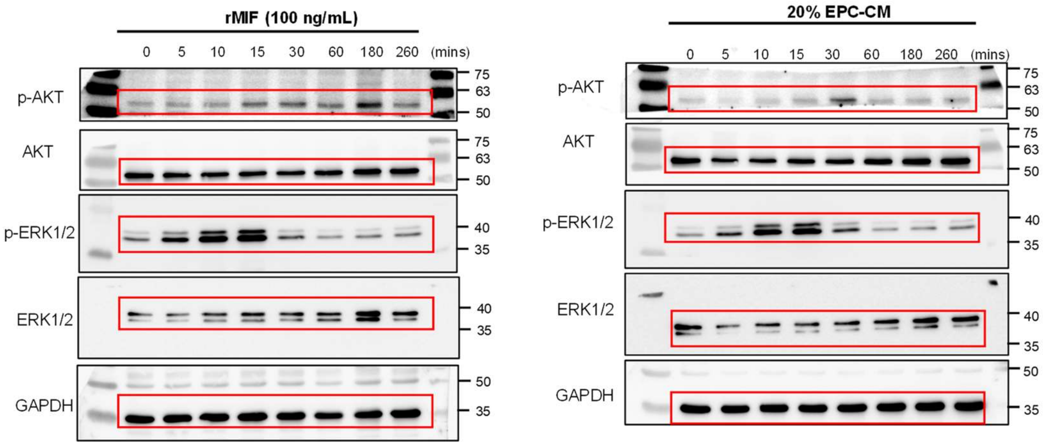

Fig. S4 Corresponding full-length immunoblot of Figure 6A

Western blot analysis of p-AKT, AKT, p-ERK1/2, ERK1/2, and GAPDH in Figure 6A. The red rectangles indicate the cropped immunoblot.

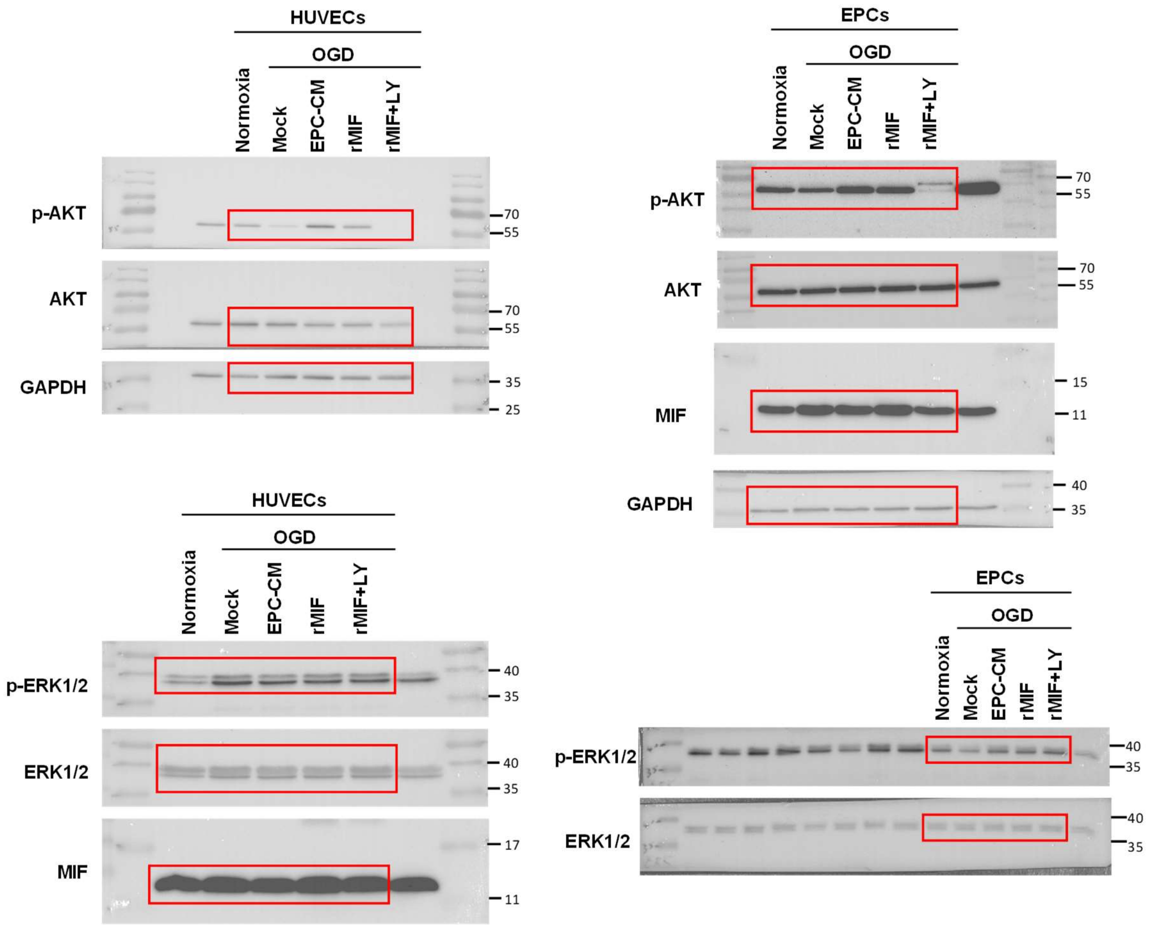

Fig. S5 Corresponding full-length immunoblot of Figure 6B

Western blot analysis of p-AKT, AKT, p-ERK1/2, ERK1/2, MIF, and GAPDH in Figure 6B. The red rectangles indicate the cropped immunoblot.
